# From immersion to burnout: anxiety mechanisms and pathways to motivational exhaustion in gamified health education

**DOI:** 10.3389/fpubh.2026.1732924

**Published:** 2026-02-04

**Authors:** Yangyang Lin, Chuyan Mo, Baojian Wei

**Affiliations:** 1 School of Smart Health Care, Zhejiang Dongfang Polytechnic, Wenzhou, Zhejiang, China; 2 Faculty of Business, The University of Hong Kong, Hong Kong, China; 3 School of Nursing, Shandong First Medical University & Shandong Academy of Medical Sciences, Tai'an, Shandong, China

**Keywords:** anxiety, digital literacy, gamified learning, health education, immersive experience, learning burnout, motivational depletion, psychological sustainability

## Abstract

Gamified health education is increasingly used to sustain learners’ motivation, yet its underlying psychological mechanisms remain insufficiently understood. Drawing on self-determination theory, flow theory, and resource-depletion perspectives, the present study employed a cross-sectional survey to examine how immersion, anxiety, and motivational depletion jointly shape learning burnout in gamified health education contexts. Data were collected from 350 university students who reported regular engagement with gamified health learning activities. Structural equation modeling revealed that immersion was negatively associated with anxiety (*β* = −0.45, *p* < 0.001) but indirectly related to higher burnout through motivational depletion (indirect *β* = −0.14, *p* < 0.001). Anxiety mediated the pathway between immersion and depletion (*β* = 0.52, *p* < 0.001) and also directly predicted burnout (*β* = 0.25, *p* = 0.001). Polynomial regression further identified an inverted-U relationship between immersion and anxiety, indicating that moderate engagement stabilizes emotional experience, whereas excessive immersion generates psychological tension. Latent profile analysis distinguished three learner groups—resilient, stable, and high-risk—and moderation analysis showed that digital literacy buffered the association between depletion and burnout (*β* = −0.12, *p* = 0.006). Taken together, these findings suggest that engagement and exhaustion unfold within the same motivational system. Building on this pattern, the study advances the concept of psychological sustainability, emphasizing that effective gamified learning depends not only on motivational activation but also on opportunities for recovery. Accordingly, gamified health education should incorporate adaptive challenge, reflective pauses, and emotional regulation supports to sustain engagement without depleting psychological resources.

## 1 Introduction

With the rapid integration of digital health and educational technologies, gamification has moved beyond an experimental design approach to become a routine strategy in global health education. Rather than serving merely as an engagement tool, gamified interventions are now embedded in a wide range of practices aimed at shaping health behaviors and sustaining learning participation. Early empirical work by Johnson et al. (1) demonstrated that design elements such as points, levels, performance feedback, and competitive structures can meaningfully influence individuals’ willingness to engage. This functional effectiveness has led to the adoption of gamification across heterogeneous contexts, including health promotion, chronic disease management, and mental health interventions. As empirical research has expanded across countries and populations, findings have become increasingly nuanced. Drawing on a comprehensive scoping review, Aschentrup et al. (2) showed that gamified digital interventions can support adult mental health prevention and health promotion, yet their effectiveness varies substantially depending on intervention design, duration, and targeted psychological outcomes. In professional health education settings, van Gaalen et al. (3) reported consistent gains in short-term engagement and learner satisfaction following gamified instruction. However, evidence regarding how these designs influence cognitive load and deeper psychological processes remains fragmented and methodologically uneven. The scope of gamified health interventions has also broadened in terms of target populations. A meta-analysis conducted by Wang et al. (4) identified a moderate overall effect of gamification on physical activity among children and adolescents, suggesting that playful motivational structures can translate into measurable behavioral change. Comparable patterns have been observed in later life stages: Chen et al. (5) found that gamified mHealth interventions were associated with increased physical activity among older adults. Despite these promising outcomes, most studies prioritize behavioral indicators—such as activity frequency or adherence rates—while devoting limited attention to participants’ subjective experiences during sustained engagement. In particular, anxiety responses, perceived psychological load, and gradual motivational depletion remain underexplored, even though these processes may critically shape long-term effectiveness. This empirical emphasis on observable behavior contrasts with the theoretical evolution of gamification and immersive experience. The psychological foundations of gamification research are commonly traced to Self-Determination Theory (6), which conceptualizes intrinsic motivation as emerging from the fulfillment of autonomy, competence, and relatedness needs. This framework has been instrumental in explaining why gamified environments initially activate engagement. Subsequent theoretical developments shifted attention from motivation alone to the quality of experience. Flow Theory (7) conceptualizes deep immersion as a state arising from the dynamic alignment between perceived challenge and individual skill, highlighting experiential intensity rather than motivational input. As digital environments became increasingly complex and immersive, Slater and Wilbur (8) extended the concept of immersion by emphasizing the role of technological affordances in shaping psychological involvement through a sense of presence. Alongside these experience-oriented perspectives, scholars have raised concerns regarding the limits of cognitive and psychological resources. Cognitive Load Theory (9) explicitly argues that learning is constrained by working memory capacity, implying that prolonged or overly demanding stimulation may undermine rather than enhance learning outcomes. From this perspective, immersion represents a conditional advantage rather than an inherently positive state. Building on these insights, O’Brien and Toms (10) proposed a process-oriented framework of user engagement, defining engagement as a dynamic trajectory characterized by continuous regulation across cognitive, emotional, and behavioral dimensions. Nevertheless, empirical research on gamification has largely retained a short-term activation focus, offering limited insight into longer-term psychological consequences. Eysenbach’s (11) formulation of the “law of attrition” captures the widespread decline in engagement observed in digital interventions, yet explanations have predominantly remained descriptive and behavior-centered. Theoretical models of self-regulation offer a potentially productive lens for addressing this gap. Baumeister et al.’s (12) strength model of self-control suggests that sustained goal pursuit and self-regulatory effort draw upon finite psychological resources, which may result in fatigue, reduced persistence, and functional impairment over time. Despite its relevance, this resource-based perspective has rarely been integrated into research on gamification or immersive learning environments. Evidence from educational contexts further underscores the need for such integration. In a longitudinal classroom study, Hanus and Fox (13) observed that while gamification enhanced engagement in early stages, it was later associated with declines in intrinsic motivation and heightened social comparison pressure. Importantly, their findings point to motivational erosion rather than simple disengagement, yet the psychological mechanisms underlying this shift were not systematically examined. Complementary work by Perski et al. (14) and Yardley et al. (15) similarly argues that engagement should be conceptualized as an evolving regulatory process involving cognitive investment, emotional responses, and behavioral maintenance, rather than as a static measure of usage intensity. Despite these advances, the interrelations among immersive experience, anxiety responses, and psychological resource depletion remain theoretically fragmented. Addressing this gap, the present study adopts a psychological sustainability perspective to examine how immersive experience in gamified health education interacts with anxiety mechanisms and motivational depletion across sustained participation. By focusing on these dynamic processes, this study seeks to clarify why gamified interventions support learning and health education in some contexts, yet contribute to motivational decline in others.

## 2 Literature review

### 2.1 Gamified health education: research progress

Over the past decade, gamified health education has expanded rapidly within both education and public-health domains, emerging as an approach that connects digital technology with learning motivation. A bibliometric analysis by Yıldız et al. (16) documents a steep upward trajectory since 2010, with most research originating in Europe, North America, and East Asia. However, growth in volume has not been matched by equivalent theoretical or methodological maturity. Topic distributions show clear “hot spots” and fragmentation, suggesting that the field, while interdisciplinary in form, still lacks conceptual depth and coherent explanations of underlying mechanisms. A more integrated empirical picture appears in the work of Bryant, Sisk, and McGuire (17), whose systematic review and meta-analysis indicate that gamified digital interventions can significantly improve self-efficacy and anxiety management among children and adolescents. Yet the results vary widely depending on program length, design features, and learner characteristics. Similarly, Bas-Sarmiento et al. (18) report that although gamified interventions tend to produce short-term gains, inconsistent methods, culturally narrow samples, and limited follow-up reduce claims of durability. These findings together imply that gamification is not simply a “motivational booster” but a complex psychosocial system in which activation and exhaustion coexist. Within the broader health education landscape, medical education has served as a major testing ground. Drawing on the SOLO taxonomy, Huang et al. (19) show that most gamified initiatives focus on lower-order cognitive outcomes such as factual recall and skill acquisition, while evidence for effects on higher-order competencies—clinical reasoning, collaborative decision-making, or empathy—remains inconclusive. Singhal et al. (20) offer practical design guidelines emphasizing playfulness, feedback, and continuous participation, though these remain grounded more in instructional experience than theoretical validation. Using a Delphi approach, Wang et al. (21) identify priority gamification elements—immediate feedback, social comparison, and goal-setting—but the literature still provides limited understanding of how these features operate through psychological processes to influence motivation, anxiety, and potential burnout. Overall, research in medical education remains strongly design-driven rather than mechanism-driven, leaving theoretical development relatively shallow. From a psychological perspective, the studies by Cheng (22) and Castellano-Tejedor (23) mark an important turn toward integrating gamification and mental-health research. Cheng finds that gamification can strengthen positive affect and a sense of accomplishment, while also noting that its reward structures may heighten anxiety sensitivity—a “double-edged” effect. Castellano-Tejedor similarly argues that affective activation through competition and feedback can enhance engagement but may also contribute to fatigue and motivational decline. These studies highlight the potential psychological costs of sustained participation, including the gradual depletion of emotional energy and diminished feelings of control. Longitudinal evidence reinforces these concerns. Spahl’s (24) review of adolescent interventions shows that while early stages of gamified learning often improve health behaviors, participation tends to drop during extended follow-ups, with fatigue and boredom emerging as frequent complaints. This pattern illustrates the fragility of motivational maintenance and underscores the need for longitudinal and dynamic measures that capture how engagement transforms into exhaustion over time. Empirical confirmation of this process is provided by Altmeyer et al. (25), who conducted a multi-month experiment in a gamified fitness-learning setting. Their study reveals a clear “motivation-decay curve”: personalization and competitive feedback initially boost participation but later generate anxiety and strain as novelty diminishes. This finding challenges assumptions of uniformly positive outcomes, demonstrating that motivational enhancement and psychological depletion can coexist within a single system. It also establishes a temporal framework for modeling the pathway from immersion to anxiety, fatigue, and eventual burnout. Viewed collectively, these studies chart the evolution of gamified health education from its conceptual beginnings to its diffusion across multiple domains, while exposing persistent theoretical and practical tensions. Much of the existing research remains anchored in technological design, with insufficient attention to the psychological mechanisms that sustain or undermine motivation. Most studies emphasize short-term outcomes and neglect the gradual erosion of psychological energy. Furthermore, limited attention to cultural and educational variation restricts generalizability beyond high-income contexts. Advancing this field will require moving beyond metrics of participation to examine how gamification reorganizes learners’ psychological resources and emotional balance over time, enabling a more sustainable and human-centered approach to health education.

### 2.2 The dual psychological effects of immersion

Immersive experience has increasingly been recognized as a psychologically consequential dimension within gamified learning and health education, particularly in relation to learners’ motivational engagement, anxiety regulation, and perceived energy expenditure. Rather than functioning as a uniformly beneficial mechanism, immersion appears to operate through context-dependent psychological pathways whose effects unfold differently across time, task demands, and individual regulatory capacities. Empirical evidence supporting the short-term psychological benefits of immersive design can be found in the study by Carcelén-Fraile et al. (26), who examined the effects of active gamification among primary school children. Their findings indicated reductions in anxiety levels and improvements in sleep quality, which the authors associated with enhanced attentional focus and positive affect elicited by embodied feedback and immediate performance-related rewards. Importantly, however, these effects were observed within relatively brief intervention windows, and the study design did not allow for the examination of longer-term psychological trajectories or inter-individual variability. As a result, it remains unclear whether the observed benefits reflect enduring changes in emotional regulation or context-specific responses to novelty and situational engagement. Related concerns emerge in clinical applications of immersive technologies. Sánchez-Caballero et al. (27), in their systematic review of immersive virtual reality interventions in pediatric and adolescent medical contexts, reported consistent reductions in procedural pain and anxiety. At the same time, their analysis revealed substantial variability in effect sizes, which appeared to depend heavily on interaction design features, including the degree of sensory immersion and perceived realism. Notably, interventions characterized by higher levels of sensory intensity did not invariably produce stronger psychological benefits. In some cases, excessive immersion was associated with attentional fragmentation and emotional fatigue, suggesting that immersive experience may be subject to threshold effects rather than linear enhancement. This non-linear interpretation is further supported by broader reviews of immersive technologies in medical education. Iqbal et al. (28) highlighted the capacity of virtual and augmented reality tools to enhance presence, empathy, and procedural accuracy, yet cautioned against an uncritical reliance on technological richness. They observed that highly scripted or visually dominant immersive environments may inadvertently constrain learners’ autonomous exploration and reflective processing. A similar pattern was identified by Lampropoulos et al. (29), whose review of immersive tools in medical and nursing education showed that increased engagement during training sessions did not consistently translate into sustained competence or confidence. In some instances, learners reported heightened performance pressure and learning-related anxiety, particularly when immersive tasks were cognitively demanding or evaluative in nature. Evidence from higher education contexts reinforces this ambivalence. Park et al. (30), in a meta-analysis of immersive teaching interventions for nursing undergraduates, reported improvements in learning satisfaction and short-term knowledge retention. However, these effects tended to attenuate over time, and several primary studies included in the analysis documented experiences of perceived overload and emotional withdrawal. On this basis, the authors proposed the immersion threshold hypothesis, suggesting that immersive experiences may become psychologically counterproductive when task demands exceed learners’ regulatory or attentional resources. While this hypothesis remains in need of longitudinal validation, it aligns with earlier findings from long-term gamification research indicating that sustained high-intensity engagement can contribute to motivational decline and burnout (25). Design-oriented research offers partial strategies for addressing these tensions. Zhang and Zhang (31), examining co-designed immersive well-being technologies developed in collaboration with healthcare professionals, found that participatory design processes helped moderate psychological load by aligning technological complexity with users’ interpretive frameworks and professional practices. Their findings suggest that the psychological benefits of immersion depend less on sensory intensity than on users’ sense of agency and their ability to integrate immersive experiences into meaningful cognitive and emotional narratives. The social dimensions of immersion introduce additional complexity. Otani and Djamnezhad (32) noted that immersive social interactions can strengthen feelings of connectedness and shared identity, yet may simultaneously heighten social anxiety and self-evaluative concerns, particularly in competitive or comparison-driven environments. From a related perspective, Pragya and Biswajita (33) introduced the concept of situational empathy effects in augmented reality contexts, arguing that immersive experiences can produce emotional misalignment and cognitive dissonance when users are exposed to affectively intense but contextually incongruent stimuli. Although their analysis focused on commercial applications, the underlying psychological mechanisms are relevant to health education settings, where emotional engagement must be carefully calibrated to avoid psychological exhaustion. Taken together, these studies suggest that immersive experience in health education cannot be adequately understood as either inherently beneficial or inherently detrimental. While multisensory interaction and real-time feedback may support attention, emotional engagement, and short-term motivation, poorly calibrated or overly intensive immersive designs risk undermining learners’ sense of control and psychological sustainability. Rather than maximizing immersion per se, future research should focus on identifying context-sensitive design parameters and individual difference factors that shape how immersive experiences are processed over time. Such an approach would enable more precise theoretical models capable of explaining when and for whom immersion supports learning and well-being, and when it contributes to anxiety, overload, or motivational depletion.

### 2.3 Anxiety mechanisms and motivational depletion pathways

Within gamified health education, anxiety and motivational exhaustion play a decisive role in explaining how learners sustain participation and manage psychological energy. Anxiety often signals rising motivation, yet it can just as easily mark the onset of fatigue. This shifting function makes it a central process for understanding how intense engagement may eventually turn into burnout. From a cognitive–behavioral perspective, Curtiss et al. (34) describe anxiety as more than a single emotional reaction. It reflects the interaction of how individuals interpret a situation, their physiological arousal, and their sense of competence. Cognitive-behavioral therapy (CBT) helps to reduce anxiety by identifying and revising negative thinking patterns, providing a useful way to understand how cognition, emotion, and behavior influence one another in gamified environments. Still, CBT-based strategies were designed primarily for clinical use and may not fully account for anxiety that arises from performance pressure or from the constant feedback built into digital learning systems. Fumero et al. (35), in their review of mindfulness-based interventions, suggest that the regulation of anxiety depends largely on how attention is managed. Mindfulness strengthens awareness of mental states and limits the cognitive drain caused by worry, allowing individuals to redirect effort toward learning. This interpretation implies that managing anxiety involves the redistribution of attention as much as emotional control. However, methods that work well in controlled or therapeutic contexts often struggle to adapt to the fast, feedback-intensive nature of gamified learning. When systems deliver rewards or updates in rapid cycles, anxiety may be reactivated faster than mindfulness can offset it. Li et al. (36) examine similar processes in digital health apps from an information-systems perspective. They find that users’ motivation, initially stimulated by rich feedback, tends to shift toward dependence on external cues. As feedback slows or rewards lose novelty, engagement becomes passive, and emotional fatigue increases. Anxiety about maintaining progress gradually gives way to exhaustion—a process Li and colleagues describe as an “anxiety–dependence–depletion” cycle. Their conclusions are consistent with Livermon et al. (37), who observed comparable motivational decline among students and staff using mobile psychological intervention programs. Even digital systems that initially reduce anxiety may, over time, create new forms of strain. When platforms emphasize frequent check-ins or constant self-monitoring, users sometimes develop what researchers call “psychological fatigue” or “digital resistance,” marked by avoidance of feedback and declining willingness to self-evaluate. This shift reveals a kind of reverse reinforcement: feedback that once sustained participation begins to generate stress and diminish energy. Nahum-Shani et al. (38) describe this as the engagement paradox. External incentives can raise activity levels at first, but if internal motivation does not grow alongside them, participation becomes shallow and emotionally unstable. They argue that the success of digital interventions should be measured not only by short-term engagement but also by how effectively they balance psychological resources with behavioral outcomes. This insight seems especially relevant to gamified health education. When incentive systems exceed learners’ emotional limits, high activity may conceal an underlying motivational drain. Building on these concerns, Cheng (22) proposes that the foundations of health gamification should move away from behaviorist reward models and toward approaches grounded in affective computing and psychological sustainability. In this view, anxiety is not simply a negative reaction; it can function as a manageable motivational signal. Properly calibrated challenge or time pressure may evoke achievement-oriented anxiety that enhances focus and effort. Yet without recovery mechanisms—such as intermittent rest, supportive feedback, or empathic communication—this same anxiety can accumulate and turn into depletion. Viewed together, these studies reveal a dynamic relationship between anxiety and motivational exhaustion. Anxiety emerges both as a product of external incentives and as a signal of declining internal energy. Earlier research, such as that by Curtiss and Fumero, focused on emotional regulation and cognitive restructuring in clinical contexts. More recent work by Li et al. shifts attention to digital learning, tracing how anxiety evolves under conditions of continuous interaction and feedback. Still, most existing models treat the relationship as one-directional—either anxiety reduces motivation, or feedback relieves anxiety—without recognizing its reciprocal and nonlinear nature. Moreover, factors such as self-efficacy, emotional control, and digital literacy, which likely influence how individuals experience anxiety within gamified settings, remain insufficiently examined. Future studies would benefit from developing an integrated framework that conceptualizes anxiety as a regulator within the broader flow of psychological energy. This framework should explore how feedback frequency, goal evaluation, and self-monitoring interact to determine whether motivation is sustained or depleted. Such an approach could deepen theoretical understanding of gamification’s psychological mechanisms and guide the design of learning environments that support motivation while minimizing anxiety over time.

### 2.4 Integrated models of energy depletion and learning burnout

Learning burnout and energy depletion are significant concerns in digital learning environments, especially during periods of intense or prolonged study. In these settings, learners must manage, expend, and replenish their cognitive and emotional resources. Hobfoll’s (39) Conservation of Resources (COR) theory offers an insightful framework, suggesting that burnout stems from the ongoing depletion of psychological resources under prolonged stress, coupled with insufficient recovery opportunities. Schaufeli et al. (40) expanded on this idea, exploring the relationship between burnout and engagement. They argued that burnout not only involves disengagement but also encompasses emotional exhaustion, cynicism, and diminished efficacy. This process is particularly relevant in high-demand environments, including digital learning contexts. Here, continuous feedback and external rewards can further drain emotional and cognitive resources, heightening the risk of burnout. Zimmerman’s (41) Self-Regulated Learning (SRL) theory, on the other hand, highlights how learners can proactively manage their energy by setting goals, tracking progress, and adjusting strategies, thus mitigating the risk of burnout. Consequently, learning designs should provide ample flexibility, allowing learners to regulate their pace and recover as necessary. In the context of digital learning and gamification design, Deterding (42) advocates for aligning challenges with learners’ skills to foster intrinsic motivation. However, when feedback and incentives are too frequent, learners may shift from intrinsic to extrinsic motivation, potentially exacerbating emotional fatigue and cognitive overload. Therefore, careful attention must be paid to the balance between challenge and recovery to maintain motivation and emotional resilience in gamified learning environments. Sweller et al.’s (43) Cognitive Load Theory underscores the detrimental effects of excessive cognitive load, noting that overburdening cognitive resources leads to fatigue and reduced learning outcomes. In immersive learning environments, the overload of stimuli and feedback can increase cognitive load, diminishing opportunities for recovery and, ultimately, affecting learners’ well-being. To sum up, burnout and energy depletion are not static issues but dynamic processes that evolve in response to external challenges and individual regulation. In high-intensity learning tasks, it is essential for learners to manage the balance between external demands and their need for recovery. This study explores how optimal design elements in digital learning—particularly in gamified and immersive contexts—can help learners maintain a sustainable rhythm, balancing challenge and recovery, and ultimately reducing the risk of burnout.

## 3 Methods

### 3.1 Research design and theoretical framework

This study adopted a cross-sectional survey design to examine the psychological mechanisms linking immersion, anxiety, motivational depletion, and learning burnout in gamified health education contexts. Data were collected through an online questionnaire using a combination of convenience and snowball sampling to ensure representation across genders and academic years. A total of 350 valid responses were obtained from undergraduate students (175 men and 175 women). The sample included students from all four academic years, comprising 100 seniors (28.6%), 91 sophomores (26.0%), 86 first-year students (24.6%), and 73 juniors (20.9%). Participation was voluntary and anonymous, and the average completion time ranged from 5 to 10 min. Responses exhibiting clear patterns of inattentive or anomalous answering were excluded prior to analysis. The survey instrument was theoretically grounded in immersion theory, self-determination theory, and models of academic burnout, and was designed to capture key psychological processes involved in gamified learning. The instrument comprised 14 dimensions across three higher-order constructs. Immersion was operationalized through time distortion, focused attention, and enjoyment. Anxiety was assessed using items reflecting performance anxiety, social-comparison anxiety, and control-related anxiety. Motivational depletion was measured through indicators of declining autonomous motivation, loss of control, psychological fatigue, emotional exhaustion, reduced efficacy, and cynical disengagement. All items were rated on a five-point Likert scale ranging from 1 (strongly disagree) to 5 (strongly agree). To ensure content validity and clarity, the questionnaire was reviewed by subject-matter experts and refined through a pilot test prior to formal data collection. Internal consistency and construct validity were examined during the analysis phase. The final dataset was analyzed using SPSS and SmartPLS for descriptive statistics and structural equation modeling, enabling the examination of direct, indirect, and moderated pathways among immersion, anxiety, motivational depletion, and learning burnout.

### 3.2 Operationalization of gamified health education

In this study, gamified health education refers to health-related learning activities delivered in non-game instructional settings through the integration of commonly used game-inspired design elements. Participants reported regular engagement with course-embedded or application-based health education activities that incorporated gamification features widely adopted in Chinese university contexts, such as online health education modules, mobile learning platforms, or learning management systems enhanced with incentive and feedback mechanisms. Across these learning environments, core gamification components typically included point or score accumulation linked to task completion, progress indicators and level advancement reflecting learning milestones, and leaderboard or peer comparison functions that enabled learners to monitor relative performance. Immediate feedback mechanisms, such as performance notifications or achievement prompts, were used to reinforce task engagement, and health learning tasks were often structured as sequential challenges with clear short-term goals. In some cases, tasks were framed within simple mission-based or narrative contexts to enhance coherence and continuity across learning sessions. Within this setting, immersion was operationalized as a psychological state arising from sustained interaction with these gamification mechanisms, characterized by focused attention, enjoyment, and reduced awareness of time passage during learning. Immersion was treated as a dynamic and variable experience rather than a fixed property of any specific platform, acknowledging that its intensity may fluctuate with task demands, feedback frequency, and individual differences among learners. By defining gamified health education through shared design features instead of platform-specific implementations, the present study captures general psychological processes associated with gamified learning environments while maintaining methodological transparency and replicability.

### 3.3 Measurement framework

The study employed established measurement instruments that were adapted to the context of gamified health education to ensure validity and reliability. Four core constructs formed the basis of the measurement framework: immersion, gamified anxiety, motivational depletion, and learning burnout. Each construct included multiple subdimensions with corresponding items. All items were rated on a five-point Likert scale (1 = “strongly disagree,” 5 = “strongly agree”), with higher scores reflecting stronger expressions of the measured attribute. Item wording was mainly derived from the Flow Experience Scale (7), the Learning Burnout Scale (40), and the Academic Motivation Scale. These scales were refined through linguistic and contextual adjustments to fit the gamified health education setting. Before formal data collection, domain experts reviewed the instrument for content relevance and clarity, and a small pilot test was conducted to examine its structural consistency and cultural suitability. Table 1 summarizes the subdimensions of each construct and provides example items.

**Table 1:** Constructs and measurement indicators.

First-order construct	Second-order dimension	Example item (Likert 5-point)
Immersion	Temporal dissociation	“During gamified learning, I often lose track of time.”
	Focused attention	“When using the platform, I can concentrate fully on the task.”
	Enjoyment	“Gamified learning makes me feel excited and satisfied.”
Gamified anxiety	Performance anxiety	“I worry that I cannot achieve the scores required by the system.”
	Social comparison anxiety	“Seeing others score higher makes me feel tense or anxious.”
	Control anxiety	“I am concerned that I cannot control the outcomes of my learning.”
Motivational depletion	Decline in autonomous motivation	“I continue studying mainly just to complete the tasks.”
	Loss of control	“I feel unable to manage the pace and direction of my learning.”
	Psychological fatigue	“I feel mentally unable to keep focusing on my studies.”
Learning burnout	Emotional exhaustion	“I already feel physically and mentally drained by gamified learning.”
	Reduced efficacy	“I feel that I have achieved little in my studies.”
	Cynicism / disengagement	“I have lost interest in continuing with gamified tasks.”

## 4 Results

### 4.1 Validation of the measurement model

The measurement results in Table 2 show consistently high reliability and validity across all constructs. Cronbach’s *α* values range from 0.89 to 0.93, composite reliability (CR) from 0.92 to 0.95, and average variance extracted (AVE) from 0.68 to 0.79. All HTMT ratios are below 0.85, indicating satisfactory discriminant validity. From a statistical standpoint, the model appears highly stable. From a psychological perspective, however, this “ideal stability” may also suggest a tightly coupled system. In gamified health education, affective and cognitive reactions seem to converge rather than remain distinct, implying that immersion, anxiety, and motivational depletion may unfold as successive moments within one process rather than as independent states. The strong coherence within the immersion construct indicates that gamified mechanisms can effectively channel intrinsic motivation. When learning tasks are organized around cycles of challenge and reward, participants tend to enter a flow-like state with sustained attention and engagement. Yet such continuous activation requires ongoing investment of cognitive and emotional resources. As attention becomes extended, anxiety enters the process and transforms engagement into a gradual expenditure of mental energy. Similarly, the high reliability of motivational depletion and learning burnout does more than validate measurement quality. It also points to a form of structural fatigue. When external feedback is repeatedly tied to self-evaluation, initial engagement can give way to pressure and self-monitoring, leaving less room for recovery. In this way, short-term participation is reinforced while psychological recuperation is constrained. The consistently strong indices in Table 2 therefore deserve careful interpretation. High reliability and validity are desirable, but near-perfect coherence can also mask the lack of variation within psychological processes. Gamification may enhance persistence but, at the same time, contribute to subtle emotional strain and cognitive overload. Achieving psychological sustainability in health education requires recognizing diversity within these patterns and seeking balance—a point at which immersion motivates learning without sliding toward exhaustion.

**Table 2:** Reliability and validity test results of latent variables.

Construct	Cronbach’s α	CR	AVE	HTMT range	Evaluation
Immersion experience	0.89	0.92	0.73	0.42–0.78	Strong convergent validity
Anxiety mechanism	0.9	0.93	0.68	0.46–0.74	Good discriminant validity
Motivational depletion	0.91	0.94	0.71	0.49–0.77	High internal consistency
Learning burnout	0.93	0.95	0.79	0.52–0.82	Excellent reliability

### 4.2 Testing of the structural model

**Figure 1:**
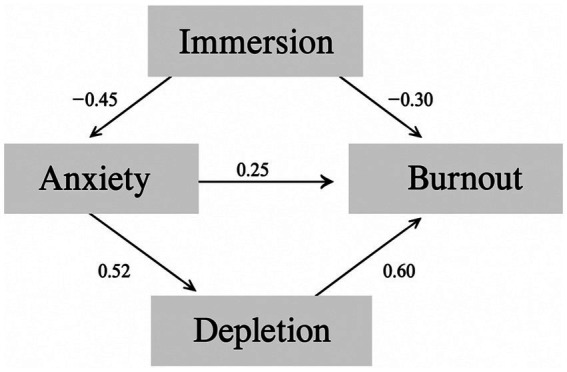
Structural equation model of the effects of immersion on burnout.

Table 3 and Figure 1 summarize the structural equation modeling results. The estimated paths show coherent and directional relations among immersion, anxiety, motivational depletion, and learning burnout. Immersion has a significant negative effect on anxiety (*β* = −0.45, *p* < 0.001). Anxiety, in turn, positively predicts motivational depletion (*β* = 0.52, *p* < 0.001), which strongly predicts learning burnout (*β* = 0.60, *p* < 0.001). Anxiety also shows a direct positive association with burnout (*β* = 0.25, *p* = 0.001), while the direct effect of immersion on burnout remains negative (*β* = −0.30, *p* < 0.001). At a descriptive level, the results suggest a sequential process in which immersion mitigates anxiety, anxiety contributes to resource depletion, and depletion increases the likelihood of burnout. Yet, from a motivational perspective, the relationships appear more dynamic than a simple linear chain. The buffering role of immersion seems to arise not merely from enjoyment but from its capacity to focus attention and absorb learners in the task. This attentional focus may temporarily protect against anxiety but also increase cognitive investment, accelerating the use of psychological resources. The strong link between anxiety and depletion implies that gamified learning can elicit performance-related tension. Continuous feedback and social comparison may heighten self-monitoring and pressure, thereby overactivating internal resources. The substantial path from depletion to burnout reflects this erosion: while external incentives sustain behavioral engagement, they may gradually weaken the internal sense of meaning, producing what can be described as a cycle of effort without renewal. The direct link between anxiety and burnout indicates that emotional strain can diminish resilience even when motivational depletion is statistically controlled, shifting the learning process from engagement toward avoidance. Taken together, the model points to a dual pattern. Immersion operates as both a driver of participation and a potential vulnerability. When challenge and feedback become overly intense, learners appear to fluctuate between stimulation and exhaustion. Gamified health education, therefore, should not be treated as a universal remedy for motivation but as a system requiring careful balance. Its effectiveness depends less on maximizing engagement than on maintaining psychological equilibrium—ensuring that immersion enhances learning without turning into a source of pressure or fatigue.

**Table 3:** Path coefficients, *t*-values, and significance levels of the structural equation model.

Path relation	Standardized β	t	*p*	Significance
Immersion → Anxiety	−0.45	9.12	< 0.001	***
Anxiety → Depletion	0.52	10.44	< 0.001	***
Depletion → Burnout	0.6	11.05	< 0.001	***
Anxiety → Burnout	0.25	3.92	0.001	**
Immersion → Burnout	−0.30	4.78	< 0.001	***

*p < 0.05 (statistically significant at the 5% level), **p < 0.01 (statistically significant at the 1% level), ***p < 0.001 (statistically significant at the 0.1% level).

### 4.3 Mediation analysis of anxiety and motivational depletion

**Figure 2:**
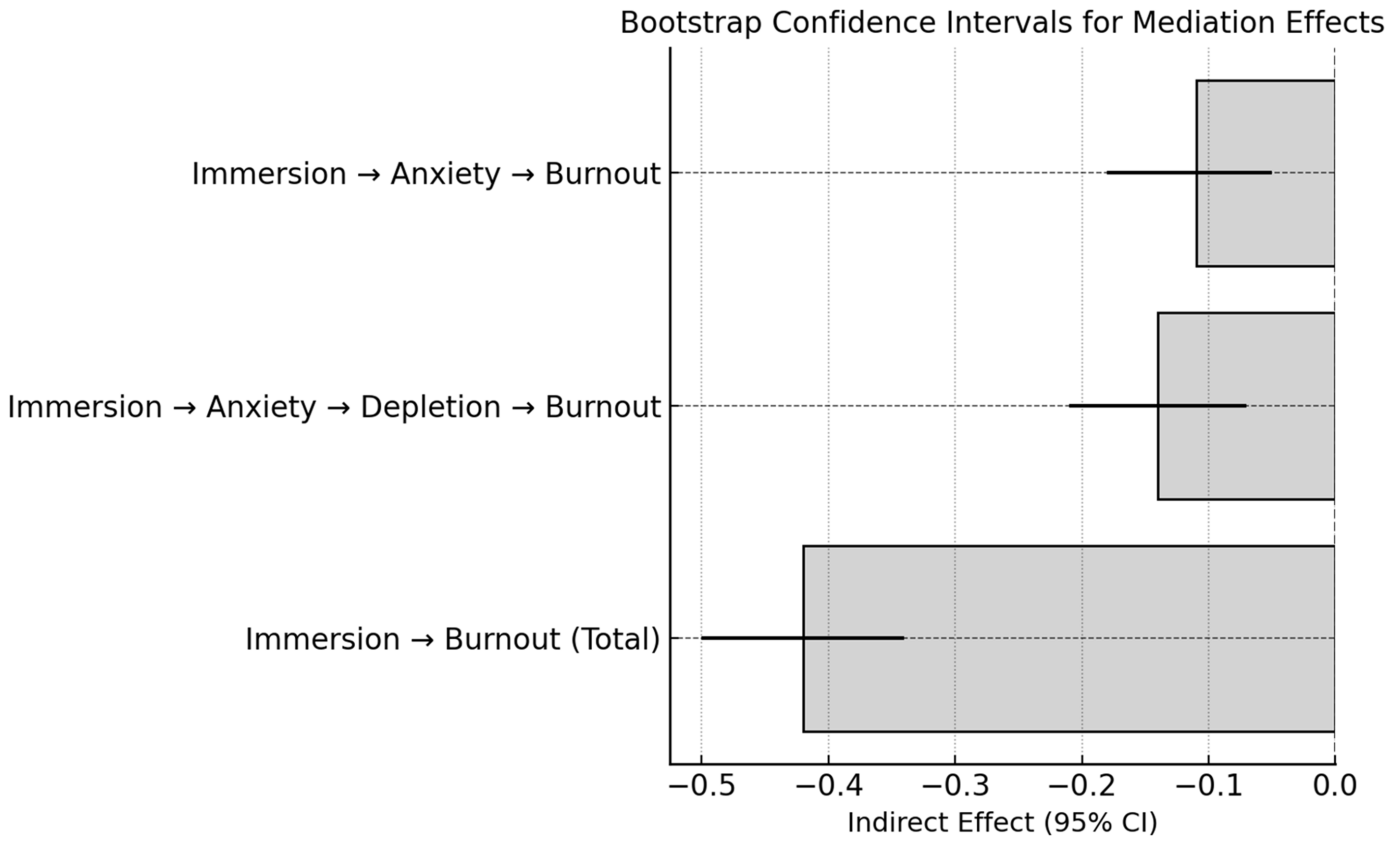
Bootstrap confidence intervals for the indirect effects of immersion on burnout via anxiety and motivational depletion.

The mediation analysis presented in Table 4 and Figure 2 depicts a system of psychological interactions marked by internal tension. Immersion shows a significant negative total effect on learning burnout (*β* = −0.42, *p* < 0.001), but this protective influence is partly reduced through two indirect routes involving anxiety and motivational depletion. The indirect path through anxiety is −0.11 (*p* < 0.01), and the sequential path through “anxiety → motivational depletion” is −0.14 (*p* < 0.001). These results suggest that immersion generally alleviates burnout, yet the same mechanism also carries counteracting tendencies. As learners concentrate attention and strive for achievement, emotional arousal increases, and anxiety becomes entangled with enjoyment. The heightened sensitivity that sustains engagement may therefore consume mental energy and narrow recovery opportunities. Motivational depletion then extends this pattern, shifting an initially absorbing experience toward fatigue. This partial offset helps explain why immersion’s benefits do not fully protect against burnout. Gamified learning depends on frequent incentives and immediate feedback that boost short-term motivation but may, over time, erode self-regulation and restorative capacity. The mediation results thus point to an uneven distribution of psychological cost: maintaining high arousal requires continuous expenditure of cognitive and affective resources. Anxiety appears as a by-product of immersion, and depletion follows as its continuation. When enjoyment and strain coexist, the intervention acquires a dual meaning. Immersion becomes both a source of engagement and a potential point of exhaustion. Theoretical discussion should therefore move beyond the significance of coefficients to address this underlying paradox. In gamified health education, motivational stimulation and emotional recovery are held in a delicate balance rather than a stable equilibrium. Designing cycles that include rest, reflection, and emotional recalibration may transform immersion from high-pressure participation into a sustainable form of engagement capable of preserving intrinsic motivation over time.

**Table 4:** Bootstrap mediation test results for the effects of immersion on learning burnout through anxiety and motivational depletion.

Mediation path	Indirect effect	95% CI	*p*	Significance
Immersion → Anxiety → Burnout	−0.11	[−0.18, −0.05]	< 0.01	**
Immersion → Anxiety → Depletion → Burnout	−0.14	[−0.21, −0.07]	< 0.001	***
Immersion → Burnout (Total Effect)	−0.42	[−0.50, −0.34]	< 0.001	***

*p < 0.05 (statistically significant at the 5% level), **p < 0.01 (statistically significant at the 1% level), ***p < 0.001 (statistically significant at the 0.1% level).

### 4.4 Moderation and group differences

The moderation and multi-group analyses in Table 5 show that individual characteristics subtly shape the pathway from immersion to burnout. Gender moderates the link between immersion and anxiety (*β* = 0.10, *p* = 0.032), digital literacy weakens the path from motivational depletion to burnout (*β* = −0.12, *p* = 0.006), and academic year exerts no measurable influence (*p* > 0.05). On the surface, these results imply minimal group-level variation. Closer inspection, however, suggests underlying differences in how learners interpret and respond to gamified learning environments. The gender pattern is unlikely to reflect purely cognitive or emotional disparities. It may instead trace back to socialized expectations that shape emotional expression and self-monitoring. Female students, often higher in affective sensitivity, may channel the arousal of immersion into anxiety more readily. Male students, in contrast, may approach gamified challenges as competitive arenas, experiencing anxiety only when performance norms are threatened. In this way, immersion becomes a culturally inflected experience rather than a uniform psychological state. The moderating role of digital literacy points to a complementary process. Learners with greater technological competence seem better able to regulate pace and manage emotional load, reducing the translation of depletion into burnout. Digital literacy thus functions less as a technical ability and more as a form of metacognitive control that supports resilience in demanding digital contexts. The absence of clear differences across academic years further suggests that psychological strain in gamified settings depends more on adaptive strategies than on age or study stage. Taken together, these findings highlight an uneven landscape of engagement. Students do not simply absorb game-based incentives but actively negotiate how immersion fits with their personal and social identities. If design strategies ignore such variation, they risk reinforcing disparities in anxiety and fatigue. Building flexibility into gamified systems—through adjustable challenge levels, reflective feedback, and autonomy-supportive structures—may allow immersion to operate as a context for individualized regulation rather than as a uniform prescription for motivation.

**Table 5:** Multi-group and moderation analysis results by gender, digital literacy, and academic year.

Moderator	Path	Δβ	*p*	Significance
Gender	Immersion → Anxiety	0.1	*p* = 0.032	*
Digital literacy	Depletion → Burnout	−0.12	*p* = 0.006	**
Grade	All paths	n.s.	*p* > 0.05	—

*p < 0.05 (statistically significant at the 5% level), **p < 0.01 (statistically significant at the 1% level), ***p < 0.001 (statistically significant at the 0.1% level).

### 4.5 Nonlinear dynamics and threshold effects of immersion

Table 6 presents evidence that immersion shows both nonlinear and threshold patterns in its associations with anxiety and burnout. The link between immersion and anxiety follows an inverted-U form (linear *β* = −0.45; quadratic *β* = 0.18, *p* < 0.01), whereas its relation to burnout remains a negative linear one (*β* = −0.30, *p* < 0.001). At lower to moderate levels, immersion appears to sharpen concentration and enhance a sense of control, thereby easing anxiety and facilitating a flow-like state. Beyond a certain point, however, cognitive and emotional load begin to rise; arousal shifts from focused engagement to tension, and anxiety increases accordingly. Deeper involvement may therefore strengthen goal pursuit while also heightening sensitivity to failure and evaluation. This inverted-U relation illustrates that the benefits of gamified learning depend on maintaining a dynamic equilibrium. When active participation drifts toward preoccupation, a constructive flow can turn into strain. The negative linear path between immersion and burnout may seem reassuring, yet it conceals the gradual costs of sustained overactivation—costs that accumulate as motivational energy and affective balance decline over time. From a practical perspective, the goal is calibration rather than maximization. Immersion functions best as a variable to be managed, not an outcome to be inflated. Designs that prioritize stimulation and reward without space for recovery risk trapping learners in a state of chronic arousal. Sustainable motivation arises when engagement is interspersed with moments of release and reflection—a rhythm of “breathable immersion” that alternates concentration with brief disengagement. When learning environments respect this rhythm, enjoyment is more likely to translate into durable and self-sustaining growth.

**Table 6:** Polynomial regression results of immersion experience on anxiety mechanism and learning burnout (nonlinear and threshold effects).

Path	Linear *β*	Quadratic β^2^	t	*p*	Significance	Pattern
Immersion → Anxiety	−0.45	0.18	2.84	< 0.01	**	Inverted U
Immersion → Burnout	−0.30	—	4.78	< 0.001	***	Linear negative

*p < 0.05 (statistically significant at the 5% level), **p < 0.01 (statistically significant at the 1% level), ***p < 0.001 (statistically significant at the 0.1% level).

### 4.6 Model robustness and alternative model comparison

Table 7 shows that the full model performs better than its simplified alternatives in explanatory and predictive strength (R^2^ = 0.64 vs. 0.49; Q^2^ = 0.47 vs. 0.29) and in overall fit (CFI = 0.964; AIC lower by 143.8). The path from motivational depletion to burnout also carries a larger effect size (f^2^ = 0.35). These statistics confirm the model’s robustness, but their value extends beyond technical adequacy. They point to a coherent psychological structure in which anxiety and motivational depletion operate as the channels through which immersion gradually transforms into burnout. The stronger fit of the full model suggests that this transition is not a straightforward decline. It develops through a sequence—heightened arousal, increased anxiety, resource loss, and eventual fatigue. When these mediating stages are excluded, the motivational dynamics collapse into a simple stimulus–response pattern that misses the emotional processes driving learning behavior. The improvements in AIC and CFI therefore represent more than numerical gains; they imply a model that captures how learners balance engagement with emotional cost over time. From a theoretical standpoint, modeling in educational psychology should not focus solely on maximizing explained variance. Its aim is to describe how psychological energy is generated, consumed, and renewed within learning systems. For gamified education, the challenge is to create designs that sustain engagement without exhausting learners—to couple incentive with recovery. In this light, the full model’s superiority is both statistical and conceptual: it illustrates that durable motivation depends less on constant stimulation than on the system’s ability to regulate its own energy and maintain equilibrium.

**Table 7:** Model robustness and comparative fit indices for competing models (R^2^, f^2^, Q^2^, AIC, and CFI).

Index	Full model	Alternative model	Δ	Interpretation
R^2^ (Burnout)	0.64	0.49	0.15	Higher explanatory power
f^2^ (Depletion → Burnout)	0.35	0.22	0.13	Stronger effect size
Q^2^ (Predictive Relevance)	0.47	0.29	0.18	Improved predictive ability
AIC	2741.6	2885.4	−143.8	Better fit
CFI	0.964	0.942	0.022	Superior fit indices

### 4.7 Latent profile analysis of immersion and burnout patterns

**Figure 3:**
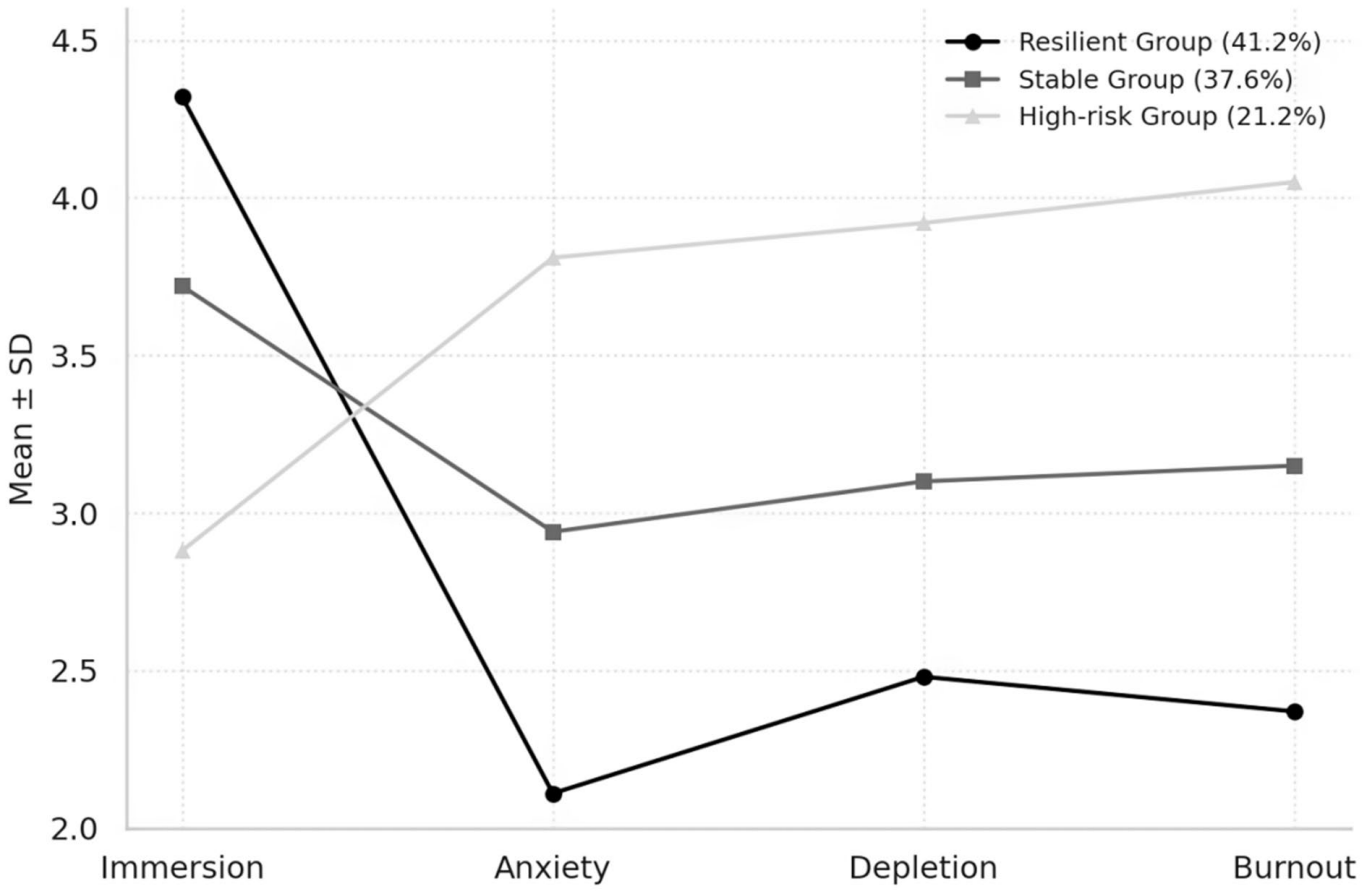
Latent profile analysis of immersion–anxiety–depletion–burnout patterns.

Table 8 and Figure 3 show clear differentiation in students’ psychological profiles within gamified health education. About 41% of participants form a resilient group—high in immersion and low in anxiety and burnout. Another 38% make up a stable group with moderate engagement and balanced resources, while roughly 21% belong to a high-risk group marked by elevated anxiety and emotional fatigue. These profiles capture more than differences in activity levels; they reflect how learners’ adaptive capacities interact with the motivational structures of gamified design. Students in the resilient group appear to retain a sense of control and personal meaning. They turn external incentives into self-driven goals and maintain flexibility even under continuous stimulation. Stable learners manage to keep motivation steady but rely heavily on feedback to stay engaged; without effective recovery mechanisms, their equilibrium is fragile and can tip toward tension when demands persist. The high-risk group is instructive for another reason: their anxiety stems not from disengagement but from insufficient psychological safety. When immersion is weak, attention wavers, and rewards begin to feel coercive rather than supportive, creating a sense of effort without agency. The distinct curves in Figure 3 visualize these contrasts. Resilient learners show relatively smooth affective patterns; the stable group exhibits mild fluctuations; and the high-risk group shows a steep downward slope of emotional energy. At a broader level, this structure exposes a paradox in gamified education: the same incentive systems that encourage participation can also intensify stress, producing divergent outcomes across learners. Designs that treat all users alike risk pushing vulnerable students into cycles of overactivation and exhaustion. A sustainable approach should therefore allow for individual pacing and reflection, enabling learners to find a workable balance between challenge and recovery rather than pursuing uniform immersion for all.

**Table 8:** Latent profile analysis (LPA) results of college students’ psychological characteristics in the immersion–anxiety–burnout model.

Profile type	% of sample	Immersion	Anxiety	Depletion	Burnout	Description
High-immersion / low-anxiety	41.2	4.32 ± 0.41	2.11 ± 0.58	2.48 ± 0.66	2.37 ± 0.61	Resilient group
Moderate / moderate	37.6	3.72 ± 0.55	2.94 ± 0.49	3.10 ± 0.52	3.15 ± 0.57	Stable group
Low-immersion / high-anxiety	21.2	2.88 ± 0.52	3.81 ± 0.44	3.92 ± 0.48	4.05 ± 0.51	High-risk group

### 4.8 Psychological network analysis

Table 9 presents a network perspective on the psychological dynamics of gamified health education. The strongest link emerges between anxiety and motivational depletion (*r* = 0.47, *p* < 0.001), followed by an even stronger association between depletion and burnout (*r* = 0.55, p < 0.001). Together they mark the main channel of psychological energy loss. Immersion correlates negatively with both anxiety (*r* = −0.39, *p* < 0.001) and depletion (*r* = −0.27, *p* = 0.002), serving as a temporary buffer rather than a lasting form of protection. Within this configuration, anxiety occupies the central node. It bridges external pressure and task engagement while accelerating the expenditure of mental resources. In gamified settings, continuous goal feedback and social comparison make anxiety ambivalent—helpful in sustaining attention yet costly when it intensifies beyond control. Once tension surpasses individual tolerance, the process moves from anxiety to depletion and finally to burnout. Immersion follows a similar dual trajectory. Initially it enhances focus and enjoyment, reducing anxiety; with prolonged arousal, it begins to consume psychological energy and erodes its own buffering capacity. The direct path between anxiety and burnout (*r* = 0.21, *p* = 0.011) indicates that excessive anxiety can spread into broader emotional functioning, leading to cumulative exhaustion. The overall structure resembles not a linear chain but a feedback loop: immersion provides short-term protection, anxiety channels external pressure, depletion carries the strain, and burnout completes the circuit. For practical application, the goal is not to eliminate anxiety but to integrate recovery into this cycle. Effective gamified design should maintain a balance between challenge and restoration, ensuring that engagement strengthens learning capacity without exhausting emotional resources and that psychological energy can circulate back into renewal rather than dissipation.

**Table 9:** Psychological network structure among immersion experience, anxiety mechanism, motivational depletion, and learning burnout variables.

Node pair	Edge weight (r)	*p*	Significance	Interpretation
Anxiety–depletion	0.47	< 0.001	***	Anxiety as core driver of energy loss
Depletion–burnout	0.55	< 0.001	***	Burnout as energy output node
Immersion–anxiety	−0.39	< 0.001	***	Immersion provides psychological protection
Immersion–depletion	−0.27	0.002	**	Indirect moderation of energy flow
Anxiety–burnout	0.21	0.011	*	Secondary propagation path

*p < 0.05 (statistically significant at the 5% level), **p < 0.01 (statistically significant at the 1% level), ***p < 0.001 (statistically significant at the 0.1% level).

## 5 Discussion

### 5.1 The paradox of engagement and exhaustion: reconstructing the psychological energy system in gamified learning

The findings of this study indicate that psychological energy dynamics in gamified learning are not linear but cyclical in nature. Immersive experience was associated with reduced anxiety (*β* = −0.45, *p* < 0.001), yet indirectly related to learning burnout through motivational depletion (*β* = −0.14, *p* < 0.001). Engagement and exhaustion therefore appear as interconnected phases within the same motivational system rather than as opposing outcomes. In the early stages of engagement, immersion supports attentional focus, strengthens perceived control, and facilitates motivational investment. When this state of heightened activation is maintained over time and repeatedly reinforced through external feedback, however, the same processes begin to draw on psychological resources rather than replenish them. This pattern is consistent with a dynamic model of psychological energy regulation. Immersion concentrates cognitive and emotional resources on task execution; anxiety influences how these resources are allocated and monitored; motivational depletion reflects the gradual erosion of available energy; and burnout marks a state of systemic imbalance. The observed inverted-U relationship between immersion and anxiety (linear *β* = −0.45; quadratic *β* = 0.18, *p* < 0.01) further illustrates that engagement operates within functional limits. Moderate immersion stabilizes emotional experience and supports effective regulation, whereas excessive immersion shifts arousal toward tension and strain. Under such conditions, greater engagement does not necessarily translate into better outcomes, as motivational intensity exceeds the learner’s capacity for recovery. The profile identified as high risk provides further insight into this imbalance. Although learners in this group reported relatively low immersion (M = 2.88), they exhibited elevated anxiety (M = 3.81) and burnout (M = 4.05). This configuration suggests that when opportunities for psychological recovery are limited, external incentives and performance cues may amplify pressure rather than enhance engagement. Gamified learning thus risks sustaining behavioral activity while simultaneously accelerating the depletion of psychological energy. Sustainable engagement depends not on maximizing stimulation but on maintaining a workable balance between challenge and restoration.

### 5.2 Anxiety as a regulatory node: the affective structure of learning sustainability

The model results suggest that anxiety plays a central role in the psychological energy system of gamified learning. It mediates the link between immersion and motivational depletion (*β* = 0.52, *p* < 0.001) and also predicts learning burnout directly (*β* = 0.25, *p* = 0.001). In this position, anxiety acts less as a symptom than as a regulatory mechanism that can both sustain and disrupt motivation. When present at moderate levels, it sharpens attention, supports goal pursuit, and strengthens self-monitoring, helping learners remain engaged. As external incentives intensify and performance feedback becomes more frequent, however, anxiety’s function changes from facilitative to constraining. Heightened vigilance gives way to tension and self-doubt, and the motivational system shifts from approach to avoidance. These patterns indicate that anxiety operates as a dynamic process rather than a fixed trait. When the learning environment maintains high arousal without opportunities for emotional recovery, the regulatory capacity of anxiety is exceeded and stress begins to dominate self-regulation. Network analysis supports this interpretation. Anxiety shows the strongest connections among variables (*r* = 0.47, *p* < 0.001), serving as the core link between resource expenditure and affective response. In this sense, anxiety organizes how energy circulates within the learning process. Motivation and emotion appear interdependent rather than separate: affect provides the conditions that sustain motivation, while gamified incentives introduce tension that can either drive or drain it. Continuous exposure to feedback, rankings, and competition creates cycles of expectation and comparison in which anxiety allocates attention and effort but also accumulates strain. Without sufficient emotional buffering, this tension transforms into depletion and eventually into burnout. The practical challenge is therefore not to remove anxiety but to manage its level and rhythm. Sustainable engagement requires a functional threshold that allows anxiety to energize motivation without overwhelming it. Introducing reflective pauses, meaning-oriented checkpoints, or affective reset moments can help learners regain balance. When regulated effectively, anxiety contributes to resilience by converting stress into focus; when unmanaged, it accelerates exhaustion. Learning sustainability depends not on eradicating anxiety but on enabling it to fluctuate and recalibrate, allowing motivation and recovery to remain in balance within the system.

### 5.3 Beyond uniform engagement: individual differences and the ethical boundaries of gamified education

Latent profile analysis reveals that the psychological effects of gamified learning are heterogeneous rather than universal and are shaped by individual characteristics. Resilient learners, who show high immersion and low anxiety, account for 41.2% of the sample and demonstrate strong emotional regulation and recovery capacity. A stable group (37.6%) maintains moderate motivation and emotional balance, while a high-risk group (21.2%) shows low immersion but elevated anxiety and burnout (Manxiety = 3.81; Mburnout = 4.05). When learning environments lack sufficient opportunities for psychological recovery or personalized support, gamification can shift from an incentive mechanism to a source of stress amplification. These findings challenge the design assumption of uniform engagement: immersion does not benefit all learners equally and may activate deeper anxiety processes in those with lower psychological resilience. Individual differences reflect not only variations in motivational intensity but also in emotion regulation, digital literacy, and socialization history. Digital literacy shows a significant moderating effect on the link between motivational depletion and burnout (*β* = −0.12, *p* = 0.006), suggesting that technically proficient learners manage workload and emotion more effectively, reducing the loss of psychological energy. These asymmetries redirect the central question of gamified education—from how to enhance motivation to whose motivation is being strengthened and whose strain is being intensified. When educational algorithms define motivation and rewards through uniform criteria, they assume psychological homogeneity and overlook variability in anxiety thresholds, affective sensitivity, and self-efficacy. Gamification thus generates new motivational opportunities but also reproduces subtle forms of inequality. Learners with greater resilience benefit from immersion, whereas those with lower emotional tolerance may be pushed toward exhaustion by the same system. This structural imbalance exposes an ethical issue in educational design. Technological systems are not neutral; their architecture carries implicit assumptions about emotion and capacity. A genuinely learner-centered approach should aim not for maximal immersion but for sustainable participation—one that respects individual limits and balances stimulation with recovery. The ethical boundary of gamified education lies not in its use but in its sensitivity to difference. Educational justice extends beyond the distribution of material resources to include the protection of psychological diversity. When learning systems make space for varying emotional rhythms and cognitive capacities, motivation can endure, and immersion can once again serve its original purpose—to support development rather than to intensify competition.

## 6 Conclusion

The present study examined the psychological processes linking immersion, anxiety, motivational depletion, and learning burnout in gamified health education using a cross-sectional sample of 350 university students. Immersion was negatively associated with anxiety (*β* = −0.45, *p* < 0.001) while also showing an indirect association with learning burnout through motivational depletion (indirect *β* = −0.14, *p* < 0.001), indicating that engagement-related processes may coexist with signs of psychological strain. Anxiety was strongly correlated with other constructs (*r* = 0.47, *p* < 0.001), linking sustained effort with the gradual erosion of motivational resources. Given the cross-sectional design, these associations should not be interpreted as causal relationships, but they suggest that activation and depletion may operate concurrently within the same motivational system. Latent profile analysis further revealed heterogeneity in learner responses, with a resilient subgroup (41.2%) maintaining relatively stable psychological functioning under higher immersion and a smaller high-risk subgroup (21.2%) reporting elevated anxiety (M = 3.81) and burnout (M = 4.05) despite lower immersion, underscoring that gamified learning does not exert uniform psychological effects across individuals. These findings point to the need to move beyond short-term engagement indicators toward a focus on psychological sustainability, understood as the regulation of motivation alongside opportunities for recovery. From a practical standpoint, design features such as adaptive task difficulty, scheduled pauses, and reflective prompts may help limit prolonged high-arousal engagement and reduce the risk of motivational depletion, and evaluation criteria for gamified learning systems should consider not only participation and performance but also learners’ capacity for recovery and longer-term psychological balance. Several limitations warrant consideration, including the cross-sectional design, which restricts temporal inference, and the reliance on self-reported measures, which may introduce common-method bias; future research employing longitudinal or experimental designs, more diverse samples, and behavioral or physiological indicators of emotional load would provide stronger tests of the proposed framework. Overall, the findings suggest that while gamified health education can support engagement, it may also impose uneven psychological demands across learners, and approaches that integrate recovery and accommodate individual variability are more likely to sustain motivation without contributing to excessive psychological burden.

### 6.1 Limitations and future directions

While this study offers theoretical and empirical insight into the psychological energy processes involved in gamified health education, several limitations should be acknowledged. The sample consisted primarily of university students, a relatively homogeneous group sharing similar academic demands and sociocultural contexts, which constrains the generalizability of the findings to other populations. Psychological processes such as anxiety, motivational depletion, and burnout are embedded in broader cultural and institutional environments, and future research should examine whether the proposed model holds across more diverse learner groups, educational settings, and cultural contexts, with particular attention to differences in social stressors, learning goals, and levels of digital literacy. In addition, the reliance on cross-sectional data limits the ability to capture temporal dynamics. Although the present design identifies associations among key variables, it cannot trace how psychological energy fluctuates, accumulates, or recovers over time. In authentic learning environments, motivation, emotion, and attention tend to unfold in cyclical patterns, and static models may obscure these processes. Longitudinal designs, experience-sampling methods, or repeated-measures approaches would allow future studies to examine how immersion, anxiety, and depletion interact across extended periods of engagement and recovery. Another limitation relates to the technological dimension of gamified learning. Psychological responses are shaped not only by individual characteristics but also by system architecture, including reward frequency, feedback timing, and interaction logic. Future work could integrate behavioral telemetry, system log data, or controlled experimental simulations to more precisely examine how specific reinforcement loops contribute to mental load and motivational depletion. Individual differences and issues of educational equity also warrant closer examination. The present findings indicate that high-risk learners experience elevated anxiety (M = 3.81) and burnout (M = 4.05) even under relatively low immersion, suggesting that vulnerability to psychological strain may emerge from limited regulatory capacity rather than excessive engagement alone. Mixed-method approaches that combine quantitative modeling with interviews, physiological indicators, or emotion-tracking techniques may help clarify how vulnerable learners navigate incentive-rich environments and why some individuals drift toward imbalance more readily than others. Addressing these limitations will support the development of gamified learning systems that are not only engaging but also attentive to recovery, individual variability, and long-term psychological balance.

## Data Availability

The raw data supporting the conclusions of this article will be made available by the authors, without undue reservation.

## References

[1] Johnson D Deterding S Kuhn K-A Staneva A Stoyanov S Hides L. Gamification for health and wellbeing: a systematic review of the literature. Internet Interv. (2016) 6:89–106. doi: 10.1016/j.invent.2016.10.002, 30135818 PMC6096297

[2] Aschentrup L Steimer PA Dadaczynski K Mc Call T Fischer F Wrona KJ. Effectiveness of gamified digital interventions in mental health prevention and health promotion among adults: a scoping review. BMC Public Health. (2024) 24:69. doi: 10.1186/s12889-023-17517-3, 38167010 PMC10763397

[3] van Gaalen AEJ Brouwer J Schönrock-Adema J Bouwkamp-Timmer T Jaarsma ADC Georgiadis JR. Gamification of health professions education: a systematic review. Adv Health Sci Educ. (2021) 26:683–711. doi: 10.1007/s10459-020-10000-3, 33128662 PMC8041684

[4] Wang M Xu J Zhou X Li X Zheng Y. Effectiveness of gamification interventions to improve physical activity and sedentary behavior in children and adolescents: systematic review and meta-analysis. JMIR Serious Games. (2025) 13:e68151. doi: 10.2196/68151, 40966596 PMC12445784

[5] Chen L Jiang F Li M Zong W Yu H. Effectiveness of mHealth-based gamified interventions on physical activity in older adults: systematic review. JMIR Aging. (2025) 8:e78686. doi: 10.2196/78686, 41171624 PMC12577663

[6] Deci EL Ryan RM. Intrinsic motivation and self-determination in human behavior. New York, NY: Plenum Press (1985).

[7] Csikszentmihalyi M. Flow: The psychology of optimal experience. New York, NY: Harper & Row (1990).

[8] Slater M Wilbur S. A framework for immersive virtual environments (FIVE): speculations on the role of presence in virtual environments. Presence Teleoperators Virtual Environ. (1997) 6:603–16. doi: 10.1162/pres.1997.6.6.603

[9] Sweller J. Cognitive load during problem solving: effects on learning. Cogn Sci. (1988) 12:257–85.

[10] O’Brien HL Toms EG. What is user engagement? A conceptual framework for defining user engagement with technology. J Am Soc Inf Sci Technol. (2008) 59:938–55. doi: 10.1002/asi.20801

[11] Eysenbach G. The law of attrition. J Med Internet Res. (2005) 7:e11. doi: 10.2196/jmir.7.1.e11, 15829473 PMC1550631

[12] Baumeister RF Vohs KD Tice DM. The strength model of self-control. Curr Dir Psychol Sci. (2007) 16:351–5. doi: 10.1111/j.1467-8721.2007.00534.x

[13] Hanus MD Fox J. Assessing the effects of gamification in the classroom: a longitudinal study on intrinsic motivation, social comparison, satisfaction, effort, and academic performance. Comput Educ. (2015) 80:152–61. doi: 10.1016/j.compedu.2014.08.019

[14] Perski O Blandford A West R Michie S. Conceptualising engagement with digital behaviour change interventions: a systematic review using principles from critical interpretive synthesis. Transl Behav Med. (2017) 7:254–67. doi: 10.1007/s13142-016-0453-1, 27966189 PMC5526809

[15] Yardley L Spring BJ Riper H Morrison LG Crane DH Curtis K . Understanding and promoting effective engagement with digital behavior change interventions. Am J Prev Med. (2016) 51:833–42. doi: 10.1016/j.amepre.2016.06.015, 27745683

[16] Yıldız M Yıldız M Kayacık AD. Rising gamification in health education: a bibliometric study. Nurse Educ Pract. (2024) 78:103993. doi: 10.1016/j.nepr.2024.103993, 38788617

[17] Bryant BR Sisk MR McGuire JF. Efficacy of gamified digital mental health interventions for pediatric mental health conditions: a systematic review and meta-analysis. JAMA Pediatr. (2024) 178:1136–46. doi: 10.1001/jamapediatrics.2024.3139, 39312259 PMC11420825

[18] Bas-Sarmiento P Julián-López C Fernández-Gutiérrez M Poza-Méndez M Marín-Paz A-J. Gamified eHealth interventions for health promotion and disease prevention in children and adolescents: a scoping review. Humanit Soc Sci Commun. (2025) 12:397. doi: 10.1057/s41599-025-04670-w

[19] Huang WD Loid V Sung JS. Reflecting on gamified learning in medical education: a systematic literature review grounded in the SOLO taxonomy 2012–2022. BMC Med Educ. (2024) 24:20. doi: 10.1186/s12909-023-04955-1, 38172852 PMC10765768

[20] Singhal S Hough J Cripps D. Twelve tips for incorporating gamification into medical education. MedEdPublish. (2019) 8:216. doi: 10.15694/mep.2019.000216.1, 38089323 PMC10712530

[21] Wang YF Hsu YF Fang KT Kuo LT. Gamification in medical education: identifying and prioritizing key elements through Delphi method. Med Educ Online. (2024) 29:2302231. doi: 10.1080/10872981.2024.2302231, 38194415 PMC10778414

[22] Cheng C. Gamification: a novel approach to mental health promotion. Curr Psychiatry Rep. (2023) 25:577–86. doi: 10.1007/s11920-023-01453-5, 37801212 PMC10654169

[23] Castellano-Tejedor C. Gamification for mental health and health psychology. Int J Environ Res Public Health. (2024) 21:990. doi: 10.3390/ijerph21080990, 39200601 PMC11353921

[24] Spahl W. Gamified digital mental health interventions for young people: a scoping review. JMIR Serious Games. (2024) 12:e64488. doi: 10.2196/64488, 39607995 PMC11638686

[25] Altmeyer M Schubhan M Krüger A Lessel P. A long-term investigation on the effects of (personalized) gamification on course participation in a gym. arXiv. (2021). doi: 10.48550/arXiv.2107.12597

[26] Carcelén-Fraile M d C Ruiz-Ariza A Rusillo-Magdaleno A Aibar-Almazán A. Effects of active gamification on sleep and anxiety reduction in Spanish primary school children. Health. (2025) 13:623. doi: 10.3390/healthcare13060623, 40150473 PMC11942101

[27] Sánchez-Caballero E Ortega-Donaire L Sanz-Martos S. Immersive virtual reality for pain and anxiety management associated with medical procedures in children and adolescents: a systematic review. Children (Basel). (2024) 11:975. doi: 10.3390/children11080975, 39201910 PMC11352374

[28] Iqbal AI Aamir A Hammad A Hafsa H Basit A Oduoye MO . Immersive technologies in healthcare: an in-depth exploration of virtual reality and augmented reality in enhancing patient care, medical education, and training paradigms. J Prim Care Community Health. (2024) 15:29241293311. doi: 10.1177/21501319241293311 PMC11528804 39439304

[29] Lampropoulos G del Bosque A Fernández-Arias P Vergara D. Virtual reality in medical education, healthcare education, and nursing education: an overview. Multimodal Technol Interact. (2025) 9:75. doi: 10.3390/mti9070075

[30] Park S Shin HJ Kwak H Lee HJ. Effects of immersive technology-based education for undergraduate nursing students: systematic review and meta-analysis using the GRADE approach. J Med Internet Res. (2024) 26:e57566. doi: 10.2196/57566, 38978483 PMC11306947

[31] Zhang Z. Zhang Z. (2025). “Beyond wellbeing apps: co-designing immersive wellbeing technologies with healthcare professionals.” *Proceedings of the ACM on human-computer interaction*, 9, 5681–5699.

[32] Otani V Djamnezhad M. Editorial: gamification, social skills, and mental health promotion. Front Psychol. (2024) 15:1441344. doi: 10.3389/fpsyt.2024.1441344, 39132314 PMC11310471

[33] Pragya J Biswajita P. The role of augmented reality in redefining e-tailing: a review and research agenda. J Bus Res. (2023) 160:113765. doi: 10.1016/j.jbusres.2023.113765

[34] Curtiss JE Levine DS Ander I Baker AW. Cognitive-behavioral treatments for anxiety and stress-related disorders. Focus (American Psychiatric Publishing). (2021) 19:184–9. doi: 10.1176/appi.focus.20200045, 34690581 PMC8475916

[35] Fumero A Peñate W Oyanadel C Porter B. The effectiveness of mindfulness-based interventions on anxiety disorders: a systematic meta-review. European J Investigation Health, Psychol Educ. (2020) 10:704–19. doi: 10.3390/ejihpe10030052, 34542506 PMC8314302

[36] Li A Sun Y Guo X Guo F Guo J. Understanding how and when user inertia matters in fitness app exploration: a moderated mediation model. Inf Process Manag. (2021) 58:102458. doi: 10.1016/j.ipm.2020.102458

[37] Livermon S Michel A Zhang Y Petz K Toner E Rucker M . A mobile intervention to reduce anxiety among university students, faculty, and staff: mixed methods study on users’ experiences. PLOS Digital Health. (2025) 4:e0000601. doi: 10.1371/journal.pdig.0000601, 39775059 PMC11706487

[38] Nahum-Shani I Shaw SD Carpenter SM Murphy SA Yoon C. Engagement in digital interventions. Am Psychol. (2022) 77:836–52. doi: 10.1037/amp0000983, 35298199 PMC9481750

[39] Hobfoll SE. Conservation of resources: a new attempt at conceptualizing stress. Am Psychol. (1989) 44:513–24. 2648906 10.1037//0003-066x.44.3.513

[40] Schaufeli WB Martínez IM Pinto AM Salanova M Bakker AB. Burnout and engagement in university students: a cross-national study. J Cross-Cult Psychol. (2002) 33:464–81. doi: 10.1177/0022022102033005003

[41] Zimmerman BJ. Becoming a self-regulated learner: an overview. Theory Into Pract. (2002) 41:64–70. doi: 10.1207/s15430421tip4102_2

[42] Deterding S. The lens of intrinsic skill atoms: a method for gameful design. Hum-Comput Interact. (2015) 30:294–335. doi: 10.1080/07370024.2014.993471

[43] Sweller J Ayres P Kalyuga S. Cognitive load theory. New York, NY: Springer (2011).

